# Osterix regulates corticalization for longitudinal bone growth via integrin β3 expression

**DOI:** 10.1038/s12276-018-0119-9

**Published:** 2018-07-18

**Authors:** Young Jae Moon, Chi-Young Yun, Hwajung Choi, Jung Ryul Kim, Byung-Hyun Park, Eui-Sic Cho

**Affiliations:** 1 Departments of Biochemistry, Chonbuk National University Medical School, Jeonju, Jeonbuk 54896 Republic of Korea; 2 Cluster for Craniofacial Development and Regeneration Research, Institute of Oral Biosciences, Chonbuk National University School of Dentistry, Jeonju, Jeonbuk 54896 Republic of Korea; 3 Departments of Orthopaedic Surgery, Chonbuk National University Medical School, Jeonju, Jeonbuk 54896 Republic of Korea; 4 Research Institute of Clinical Medicine, Chonbuk National University Hospital, Jeonju, Jeonbuk 54907 Republic of Korea

**Keywords:** Bone development, Skeleton

## Abstract

Corticalization, coalescence of trabecular bone into the metaphyseal cortex, is important for the longitudinal growth of long bones. However, little is known about the molecular mechanisms controlling corticalization. To understand the molecular mechanisms underlying corticalization, we analyzed osteoblast-specific *Osterix*-knockout mice (Col-OMT). In control mice, corticalization was initiated after 7 postnatal days, and the number of osteoblasts in the peripheral spongiosa was increased compared to the number in the central spongiosa. In contrast, in Col-OMT mice, corticalization was delayed, and the number of osteoblasts in peripheral zones was unchanged compared to the central zone. Furthermore, femoral length was decreased in Col-OMT mice at 1 month. Because Col-OMT mice exhibited impaired matrix coalescence and osteoblast migration, we evaluated integrin signaling in Col-OMT mice. Osterix bound to the *Itgb3* promoter and increased transcription of the *Itgb3* gene in osteoblast cells. Interestingly, the inner and outer cortical bones were separated in *Itgb3*-null mice at postnatal day 7. In *Itgb3*-null mice, the number of osteoblasts in peripheral zones was not changed, and the femoral length was decreased. Taken together, these results indicate that Osterix regulates corticalization for longitudinal bone growth via the control of integrin β3 expression in osteoblasts. Our findings imply that the ability to control osteoblast function during corticalization may help in the treatment of short stature.

## Bone growth: signaling for length

South Korean researchers have elucidated the molecular mechanisms that regulate bone length in mammals. Longitudinal bone growth is a tightly regulated process that mostly takes place after birth and is triggered by mechanical stimuli. Previous studies identified osterix as a key protein for bone development, but little was known about its involvement in longitudinal bone growth. Eui-Sic Cho and colleagues at Chonbuk National University in Jeonju showed that mice lacking osterix in bone-building cells (osteoblasts) have shorter limbs than control mice and that the migration of these cells to peripheral areas of bone is disrupted shortly after birth. Furthermore, they found that osterix regulates bone length by activating a gene encoding the cell adhesion protein integrinβ3. Understanding how integrin signaling in osteoblasts regulates longitudinal bone growth could lead to new treatments for short stature in humans.

## Introduction

The longitudinal growth of mammalian long bones is important in the determination of final body height and normal bone structure. The process of longitudinal bone growth is quite complex and is tightly regulated by several factors^1^. Following growth plate development, trabecular bone is formed around calcified cartilage in the ossification zone in a process called endochondral bone formation^2–5^. The newly formed trabecular bone coalesces at the metaphyseal cortex. This event is called “corticalization” and is the process by which longitudinal bone growth is completed^6^. The corticalization process during early growth is essential for the prediction of trabecular and cortical morphology in adulthood^7^. Furthermore, impaired corticalization during growth influences the fragility of the distal radius in postmenopausal women^8^. Thus, corticalization is the last stage of longitudinal growth and plays an important role in completing the structure of adult bone, though the underlying genetic and molecular mechanisms of this process remain unknown.

Longitudinal limb growth depends not only on intracellular chondrocyte signals in the growth plate but also on wide variety of mechanisms involving paracrine factors, multiple hormones, and extracellular matrix (ECM) molecules^9^. Therefore, normal variation in height cannot be defined solely by growth plate problems but must include many other genetic factors. Many studies have shown that osteoblasts and osteocytes function, in part, to regulate limb length. Mice lacking or overexpressing major bone integrity proteins exhibit longitudinal limb growth defects when compared to control mice without growth plate anomalies^10–13^. Moreover, considering that corticalization occurs after growth plate area expansion, osteoblasts and osteocytes can directly affect limb length. However, the function of osteoblasts and osteocytes has not yet been associated with longitudinal growth.

**Fig. 1:**
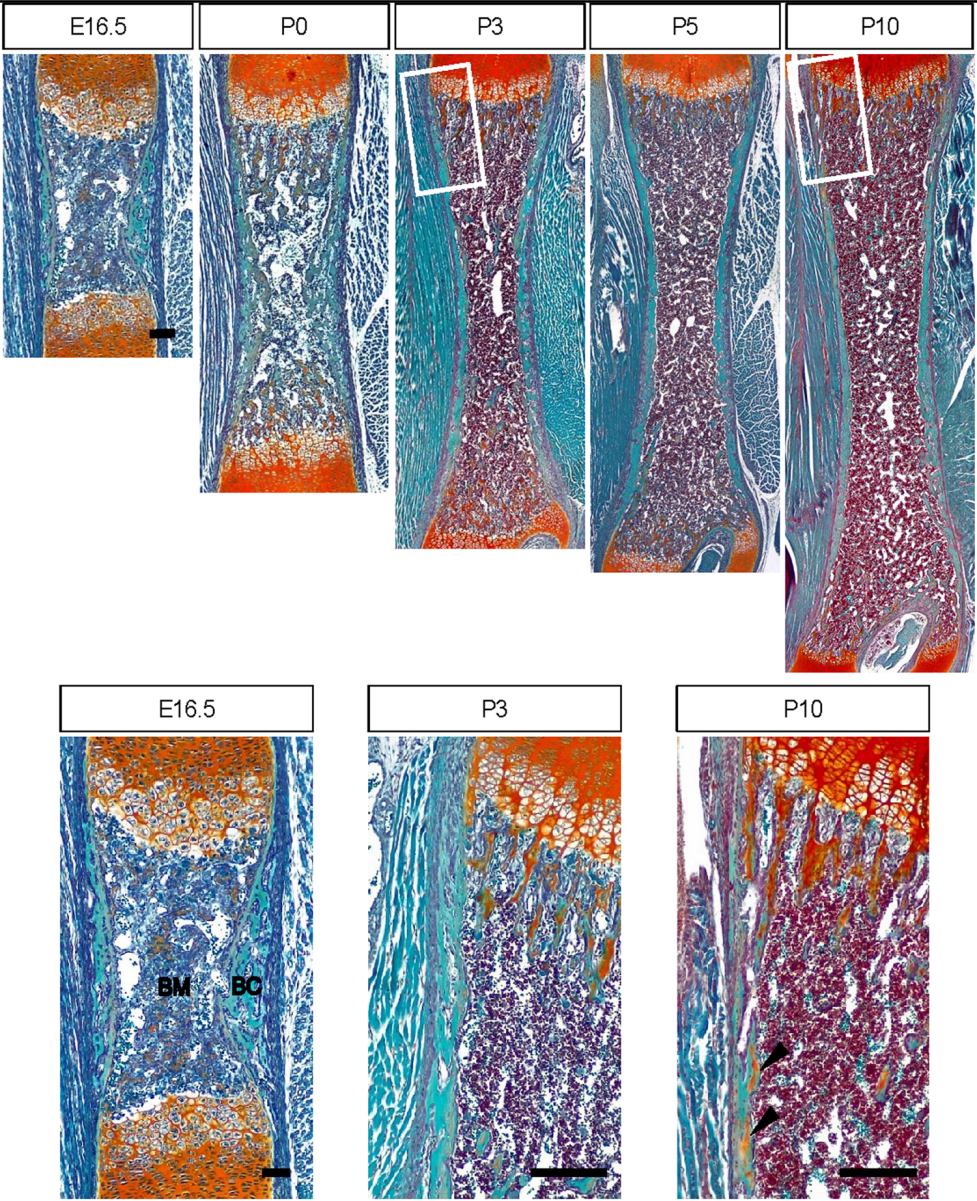
Initiation of corticalization in mouse femora. Safranin O staining of femora at E16.5, P0, P3, and P10. Black arrowheads indicate cartilage that has fused into the metaphyseal cortex. BM bone marrow, BC bone collar. Scale bar, 100 µm

During the process of endochondral bone development, the cells in the inner mesenchymal condensation compartment differentiate into chondrocytes and undergo progressive maturation into hypertrophic chondrocytes^5^. On the other hand, the outer mesenchymal condensation compartment forms the perichondrium, which directly differentiates into osteoblasts and generates a calcified bone matrix called the “bone collar” that surrounds mature chondrocytes^14^. At embryonic day 14.5 (E14.5), blood vessels invade the center of hypertrophic chondrocytes, leading to resorption of the cartilage matrix and formation of the trabecular bone matrix, which is secreted by invading osteoblast precursors and chondrocyte-derived osteoblasts^15–17^. During corticalization, trabecular bone must coalesce into cortical bone; trabecular bone is located in the inner compartment and is separated from the bone collar. After the development period, the trabecular bone will become the cortical bone in the outer compartment (Fig. 1). According to previous studies suggesting the mechanism of corticalization, mechanical stimuli and increased osteoblast surface area are both associated with corticalization^18,19^. From these proposed mechanisms and the absence of corticalization in the embryonic period, we hypothesized that osteoblast differentiation would be involved in the corticalization mechanism.

Osterix (*Osx*) is essential for osteoblast differentiation and bone formation during the embryonic and postnatal periods^20,21^. Osx also affects osteoblast migration, apoptosis, and death^22^. *Osx* deficiency in postnatal mice is associated with reductions in femoral length and thin, porous cortical bone^11,21^. In particular, trabecular bone is increased without growth plate abnormalities or defects in osteoclastogenesis. These results suggest that Osterix plays an important role in proper bone corticalization.

Corticalization is governed by mechanical stimuli^19^. Mechanical stimulation triggers signaling mechanisms that promote osteoblast proliferation, differentiation, and survival through integrin signaling^23–26^. Integrins are transmembrane molecules composed of α and β chains that bind to ECM proteins and play key roles in cell−cell and cell−ECM adhesion as well as in cell migration^27^. Those roles are analogous to the process of corticalization, in which matrix components and osteoblast in the inner compartment adhere and unify to create the outer compartment.

Here, we show the origin of corticalization over sequential time points in femora. To investigate the role of osteoblasts in longitudinal bone growth and the molecular changes that induce corticalization in osteoblasts at specific stages, we used osteoblast-specific *Osx*-knockout mice. Our results provide evidence that Osterix regulates corticalization by directly controlling integrin β3 expression.

## Materials and methods

### Animals

*Osx*^*fl/fl*^ and *Col1a1-Cre* mouse strains have been previously described^11^. *Itgb3* (B6.129S2-Itgb3^tm1Hyn^/JSemJ) mice were purchased from Jackson Laboratory (Bar Harbor, ME, USA). To generate the *Col1a1-Cre;Osx*^*fl/fl*^ (Col-OMT) mice, *Col1a1-Cre;Osx*^*fl/+*^ (control) mice were crossed with *Osx*^*fl/fl*^ mice as appropriate. *Itgb3* heterozygous mice were intercrossed to generate *Itgb3*-null mice. Mouse offspring were genotyped by polymerase chain reaction (PCR) with previously described primers^11,28^. Animals were housed in an accredited facility under a 12-h light/dark cycle and were provided water and food ad libitum. The Animal Welfare Committee of Chonbuk National University approved all protocols prior to the start of the studies.

### Histology and immunohistochemistry

Femora dissected from male mice were fixed in 4% paraformaldehyde at 4 °C overnight and decalcified in a 15% ethylenediaminetetraacetic acid solution for 2–3 weeks at 4 °C. To observe exactly the same site in each femur, femora were sectioned based on two points. The proximal point was the piriformis insertion site, and the distal point was the anterior or posterior cruciate ligament origin site. Thus, we were able to accurately obtain sections from femoral centers (Figure S1). Embedded tissues were sectioned at a thickness of 5 μm. To evaluate histologic findings, sections were stained with hematoxylin and eosin (H&E), Safranin O or picrosirius red (Sigma-Aldrich, St. Louis, MO, USA).

For immunohistochemistry, the EXPOSE Rabbit Specific HRP/DAB Detection IHC Kit (Abcam, Cambridge, MA, USA) was used according to the manufacturer’s instructions. The sections were stained using an antibody against vinculin (1:100; Abcam).

### Femoral length measurement

To accurately compare femoral lengths, we sectioned the middle part of embedded femoral tissues obtained at each time point. After H&E staining, we measured femoral length from the starting point of the distal femoral primary spongiosa to the starting point of the proximal femoral primary spongiosa, both of which were identified by stereoscopy.

### Micro-CT assessment

The femora were scanned with a micro-CT (1076 Skyscan Micro-CT, Skyscan, Kontich, Belgium) at 8-μm resolution and analyzed with CTAn software (Skyscan). A global thresholding algorithm was applied at a constant threshold for all specimens. The threshold was the intensity (gray value) that corresponded to ~50% of the average intensity of intact cortical bone. Trabecular lesions and metaphyseal cortical bone samples were analyzed in the distal metaphysis extending proximally 1.75 mm from the end of the primary spongiosa.

### Cell culture

An osteoblast cell line, MC3T3-E1, was purchased from the American Type Culture Collection (Manassas, VA, USA) and was maintained in α-MEM with 10% FBS and 100 IU/ml penicillin−100 μg/ml streptomycin at 37 °C under 5% CO_2_.

### Western blot analysis

The femora of control and Col-OMT male mice were removed from soft tissue, and the bone marrow was flushed away. Femora were frozen in liquid nitrogen and then crushed with a mortar and pestle. Then, 10 µg of protein from either bone tissue or cell lysates was separated by 10% SDS-PAGE and transferred to PVDF membranes. After blocking with 5% skim milk, the membrane was probed with a primary antibody against Osx (Santa Cruz Biotechnology), Itgb3 (Cell Signaling Technology, Beverly, MA, USA), Itgb1 (Cell Signaling Technology), p-vinculin (Abcam), GAPDH (Bioworld Technology, St Louis Park, MN, USA), and HSP90 (Enzo Life Sciences, Farmingdale, NY, USA). A horseradish peroxidase-conjugated goat anti-rabbit IgG (Enzo Life Sciences) secondary antibody was used for visualization. Signals were detected using a Las-4000 imager (GE Healthcare Life Science, Pittsburgh, PA, USA).

### Quantitative real-time PCR with reverse transcription analysis

Total RNA was extracted from dissected bone tissue using Trizol reagent (Invitrogen). RNA was precipitated with isopropanol and dissolved in diethylpyrocarbonate-treated distilled water. First-strand cDNA was generated using the random hexamer primer provided in the first-strand cDNA synthesis kit (Applied Biosystems, Foster City, CA, USA). Specific primers for each gene (Table S1) were designed using qPrimerDepot (http://mouseprimerdepot.nci.nih.gov). Each real-time RT-PCR reaction contained 1 ng of reverse-transcribed total RNA, 2 nM of forward and reverse primers, and PCR master mix and was brought to a final volume of 10 μl. RT-PCR was performed in 384-well plates using the QuantStudio™ 6 Flex Real-Time PCR System instrument (Life Technologies, Foster City, CA, USA).

### Transient transfections and reporter gene assays

Transient transfections were performed using Lipofectamine 2000 (Invitrogen) according to the manufacturer’s instructions. Briefly, 80% confluent MC3T3-E1 or HEK293 cells were transfected with empty vector (Empty) or Flag-Osx. For the reporter gene assay, we cotransfected either an empty vector or a Flag-Osx overexpression vector with the Renilla luciferase vector phRL-TK and the ITGB3-Prom-Luc reporter construct (SwitchGear Genomics, Menlo Park, CA, USA). After 48 h, cells were harvested in reporter lysis buffer (Promega, Madison, WI, USA). Luciferase activity was determined in whole cell lysates by a luciferase assay kit (Promega), and the output was expressed as relative light units. The normalized firefly luciferase signal is expressed relative to the Renilla signal.

### ChIP assay

Chromatin immunoprecipitation (ChIP) assays were performed in MC3T3-E1 cells using the SimpleChIP^®^ Enzymatic Chromatin IP kit according to the manufacturer’s instructions. The resulting extract was sonicated and used for immunoprecipitation (IP) with H3, IgG, or Osx rabbit antibodies. ChIP DNA samples were subjected to PCR using primers targeting the *Itgb3* promoter region (sense, 5′-CTGCCTCCTTAGGCTGGAAT; antisense, 5′-TAAAACTAGGGCAGGCGATG) and the *RPL30* gene (Cell Signaling Technology).

### Statistical analysis

One-way analysis of variance was used for multiple comparisons, and Student’s *t* test was used to determine significant differences between two sets of data. Differences were considered significant at *P* *<* 0.05. All data were analyzed using SPSS statistical software (version 16.0; SPSS, Chicago, IL, USA).

## Results

### Corticalization is initiated after birth in mouse long bones

**Fig. 2:**
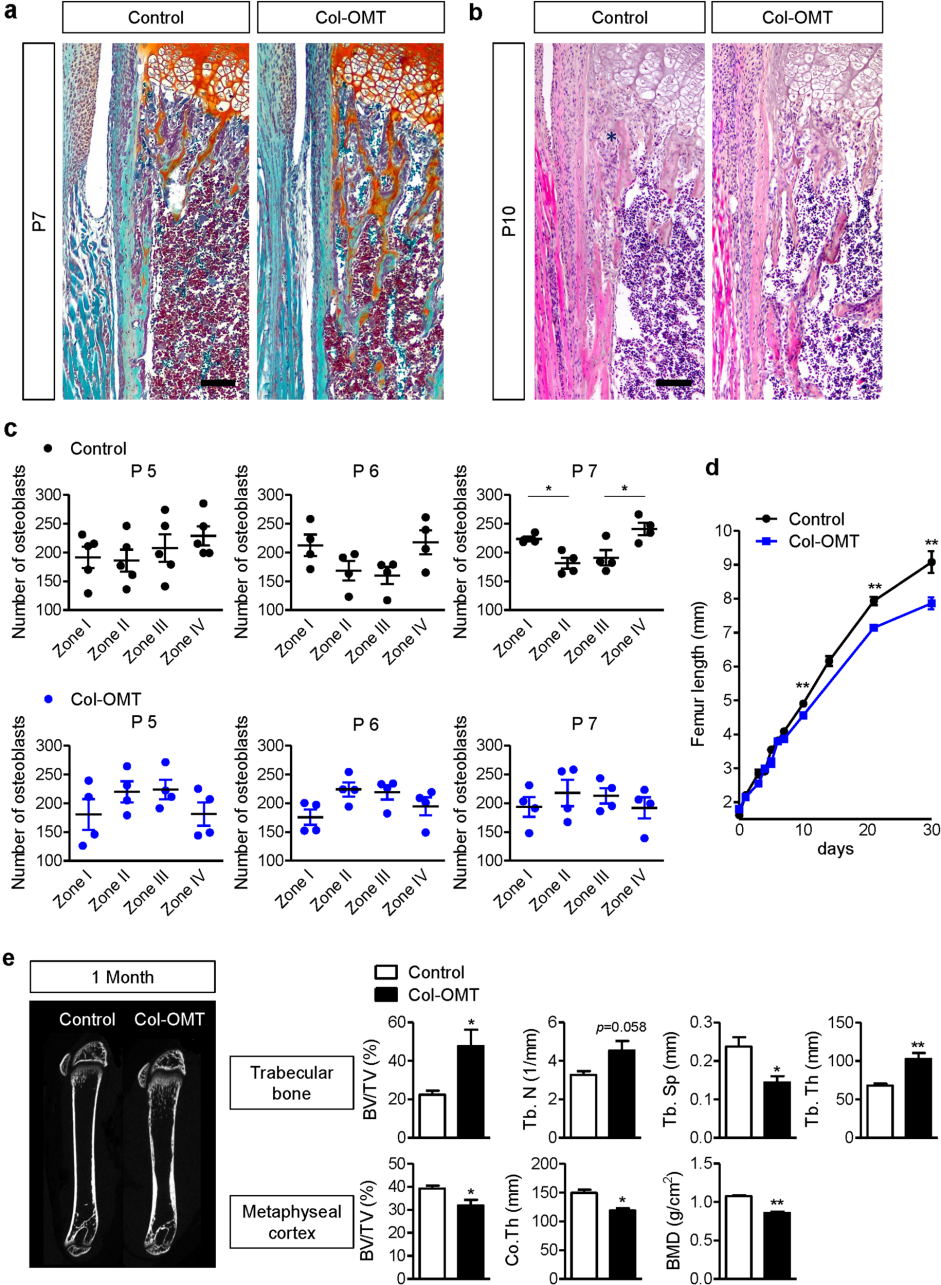
Delayed corticalization in osteoblast-specific *Osx-*knockout mice. **a** Safranin O staining of the femora of 7-day-old male littermates. **b** Hematoxylin and eosin staining of the femora of 10-day-old male littermates. Black asterisks indicate clusters of osteoblasts in the peripheral spongiosa. **c** The number of osteoblasts according to zone at P5, P6, and P7. The zones were divided into four parts (*n* = 5). Peripheral zones: zone I and IV. Central zones: II and III. Values are presented as the mean ± SEM. *p < 0.05, zone I versus zone II or zone III versus zone IV. **d** Femoral length according to age (*n* = 3–5). Femoral lengths were measured using stereoscopy after sectioning of embedded femora. **e** Morphometric indices in trabecular bone or the metaphyseal cortex of the distal femur at 1 month of age (*n* = 5). Values are presented as the mean ± SEM. *p < 0.05, **p < 0.01 versus control. Scale bar, 50 µm

During endochondral bone development, the bone collar, which is formed by intramembranous bone, and trabecular bone, which is formed by endochondral bone, are divided into different compartments. To observe the beginning of corticalization, we first subjected sequential femoral bone samples to Safranin O staining. Safranin O is a red cationic dye that binds to the anionic groups of the glycosaminoglycans (GAGs) that comprise cartilage. At E16.5, the bone collar is completely separated from cartilage-based bone material. In the initial stage after birth, trabecular bone did not coalesce with cortical bone (Fig. 1). However, coalescence of trabecular bone was observed at postnatal day 7 (P7) (Fig. 2a), and trabecular bone had obviously coalesced into the metaphyseal cortex by P10 (Fig. 1).

### Disruption of Osx in osteoblasts leads to delayed corticalization, abnormal osteoblast distribution, and decreased limb length

A previous study showed that osteoblast-specific *Osx-*knockout mice displayed both a reduction in femoral bone length and abnormal corticalization^11^. To explore the mechanisms responsible for long bone corticalization, we investigated the beginning of corticalization in the femora of osteoblast-specific *Osx*-knockout (Col-OMT) mice. First, we analyzed Cre activity using X-gal staining in *Col1a1-Cre;R26R* mice. In the distal femur of reporter mice, most osteoblasts around the trabecular bone were stained at P7, while the outer periosteum and hypertrophic chondrocytes were negative for LacZ at P7 (Figure S2). We also performed immunofluorescence to confirm the expression of Osterix in growing femora. In control mice, Osterix was localized in osteoblast nuclei in trabecular bone lesions at P7. However, the expression of Osterix was completely absent in Col-OMT mice (Figure S3). In P7 control mice, but not in P7 Col-OMT mice, the trabecular bone coalesced with the metaphyseal cortex. Further, trabecular bone volume was increased in P7 Col-OMT mice, though there was no difference in growth plate area (Fig. 2a). Because corticalization is associated with increased osteoblast surface area due to an increased number of osteoblasts in the peripheral spongiosa^18^, we analyzed osteoblast conditions in the peripheral spongiosa of control and Col-OMT mice. At P10, osteoblast clusters were observed in the peripheral spongiosa of control mice but were not observed in Col-OMT mice (Fig. 2b). To determine the difference in osteoblast distribution at the beginning of corticalization, we counted osteoblasts located in the divided spongiosa zone. In controls, although the number of osteoblasts tended to be similar in each zone at P5 and P6, the number of osteoblasts in peripheral zones (zones I and IV) significantly increased compared to central zones (zones II and III) at P7 (Fig. 2c). However, the number of osteoblasts in peripheral zones was unchanged compared to the central zones in Col-OMT mice at P7 (Fig. 2c). Next, we sought to determine when the differences in femoral bone length occurred between control and Col-OMT mice, as the rapid growth of longitudinal bones could affect the distribution of osteoblasts in controls. A difference in femoral bone length between controls and Col-OMT mice was first observed at P10. In other words, the difference in femoral bone length was observed after the change in distribution of osteoblasts and corticalization (Fig. 2d). These results demonstrate that Osterix expression in osteoblasts induces corticalization and migration of osteoblasts to peripheral spongiosa and regulates longitudinal bone growth.

In the μCT analysis of trabecular bone in the distal femur, both trabecular thickness and the bone volume to total tissue volume (BV/TV) ratio were approximately twofold higher in Col-OMT mice than in control mice at 1 month. Trabecular separation (Tb.Sp) was significantly lower in Col-OMT mice compared to control mice. On the other hand, BV/TV, cortical thickness, and bone mineral density (BMD) in the metaphyseal cortical bone of the distal femur were all significantly decreased in Col-OMT mice (Fig. 2e).

### Integrin β3 signaling is regulated by osterix in osteoblasts

**Fig. 3:**
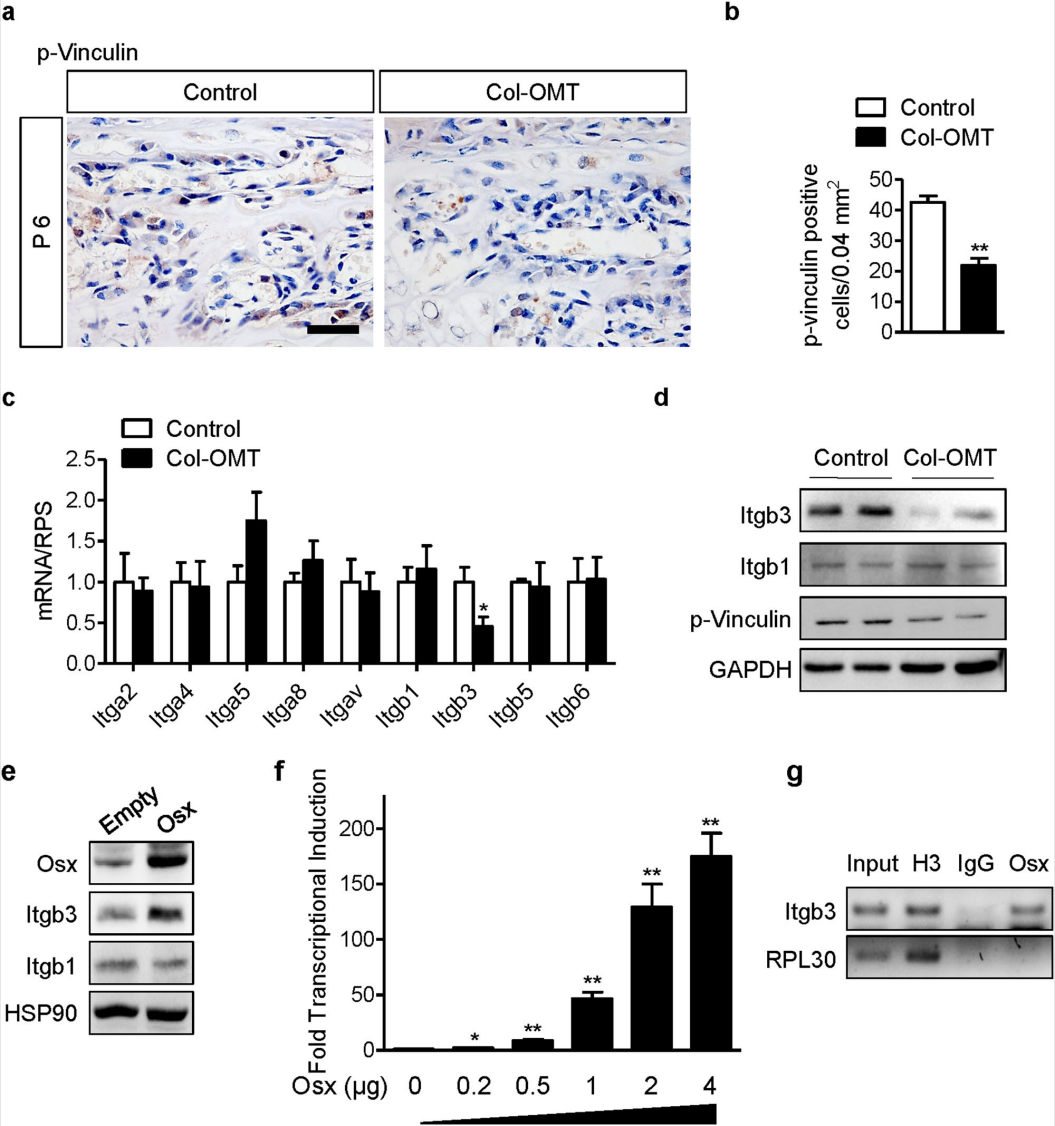
Direct regulation of integrin β3 by osterix. **a** Peripheral spongiosa lesions in the distal femora of P6 mice were immunostained with an anti-p-vinculin antibody. **b** Quantification of p-vinculin-positive cells per unit area. Values are presented as the mean ± SEM (*n* = 5). **p < 0.01 versus control. **c**, **d** Total RNA and protein was isolated from the bones of each littermate male mouse at P6. mRNA and protein levels of the indicated genes were determined by real-time RT-PCR and western blotting, respectively. Values are presented as the mean ± SEM (*n* = 5). *p < 0.05 versus controls. **e** MC3T3-E1 cells were transfected with either an empty vector or an Osx overexpression vector, and protein levels were analyzed by western blotting. **f** HEK293 cells were transiently transfected with an *Itgb3* luciferase reporter with or without increasing amounts of Osx, as indicated. Values are presented as the mean ± SEM (*n* = 6). *p < 0.05, **p < 0.01 versus control (no Osx). **g** ChIP assays were carried out in MC3T3-E1 cells. An anti-Osx antibody was used for ChIP analysis, while H3 and IgG antibodies were used as positive and negative controls, respectively. Immunoprecipitated genomic DNA was analyzed using PCR to assess protein binding to the Itgb3 promoter or a control region (RPL30). Scale bar, 25 µm

Because osteoblasts with deleted osterix could not migrate to peripheral areas and could not cluster in peripheral zones, we evaluated integrin signaling in Col-OMT mice. Integrins bind to the ECM cytoskeletal assembly and associate with signaling proteins to form focal adhesions (FAs)^29,30^. Integrin-mediated FAs composed of talin, vinculin, filamin, kindlin, tensin, and paxillin mediate cell adhesion, migration, and signaling^31^. In particular, vinculin plays a major role in stabilizing and strengthening FAs^32,33^. The phosphorylation of vinculin is required for its activation and talin-binding capacity^34,35^. Thus, we analyzed p-vinculin in osteoblasts in the peripheral spongiosa at P6. Immunohistochemical staining revealed that p-vinculin was expressed in osteoblasts, which were located in the peripheral spongiosa zone of control mice. In contrast, reduced levels of p-vinculin expression were observed in osteoblasts in the same lesion in Col-OMT mice (Fig. 3a, b). To identify the cause of reduced integrin signaling in Col-OMT mice, we analyzed the mRNA levels of integrins related to osteoblast differentiation and bone formation in P6 bone samples^23^. *Itgb3* mRNA expression levels were significantly decreased in Col-OMT mice, while no significant differences were observed in the levels of other genes (Fig. 3c). Furthermore, the protein levels of integrin β3 and p-vinculin were decreased in Col-OMT mice (Fig. 3d). These data suggest that Osx regulates integrin β3 signaling at the beginning of corticalization. To confirm if Osx could positively regulate *Itgb3* expression, MC3T3-E1 cells (osteoblast precursor cells from mouse calvaria) were transfected with an empty vector or an Osx overexpression construct. Osx overexpression was confirmed by western blotting. *Itgb3* protein levels were increased in Osx-overexpressing MC3T3-E1 cells (Fig. 3e). To investigate whether Osx transcriptionally regulates the *Itgb3* promoter, we performed cotransfection experiments in HEK-293 cells (which do not express endogenous Osx) by the use of an Osx expression plasmid and a luciferase reporter driven by the 11 kb native *Itgb3* promoter. Increasing amounts of Osx plasmid correlated with increased activity of the *Itgb3* promoter reporter (Fig. 3f), suggesting that Osx is a transcriptional activator of the *Itgb3* gene. We next performed ChIP assays to determine whether the Osx protein binds to the *Itgb3* promoter endogenously. Cross-linked and sonicated chromatin from MC3T3-E1 cells was immunoprecipitated with antibodies specific to Osx, H3 (positive control), or immunoglobulin G (negative control). The binding of immunoprecipitated proteins to the *Itgb3* promoter DNA or the control RPL30 region was determined by PCR. ChIP demonstrated that Osx specifically binds to the endogenous *Itgb3* promoter. By contrast, Osx did not bind to the endogenous control region within MC3T3-E1 chromatin (Fig. 3g). These results indicate that the *Itgb3* gene is a direct transcriptional target of Osx.

### Integrin β3 regulates corticalization

**Fig. 4:**
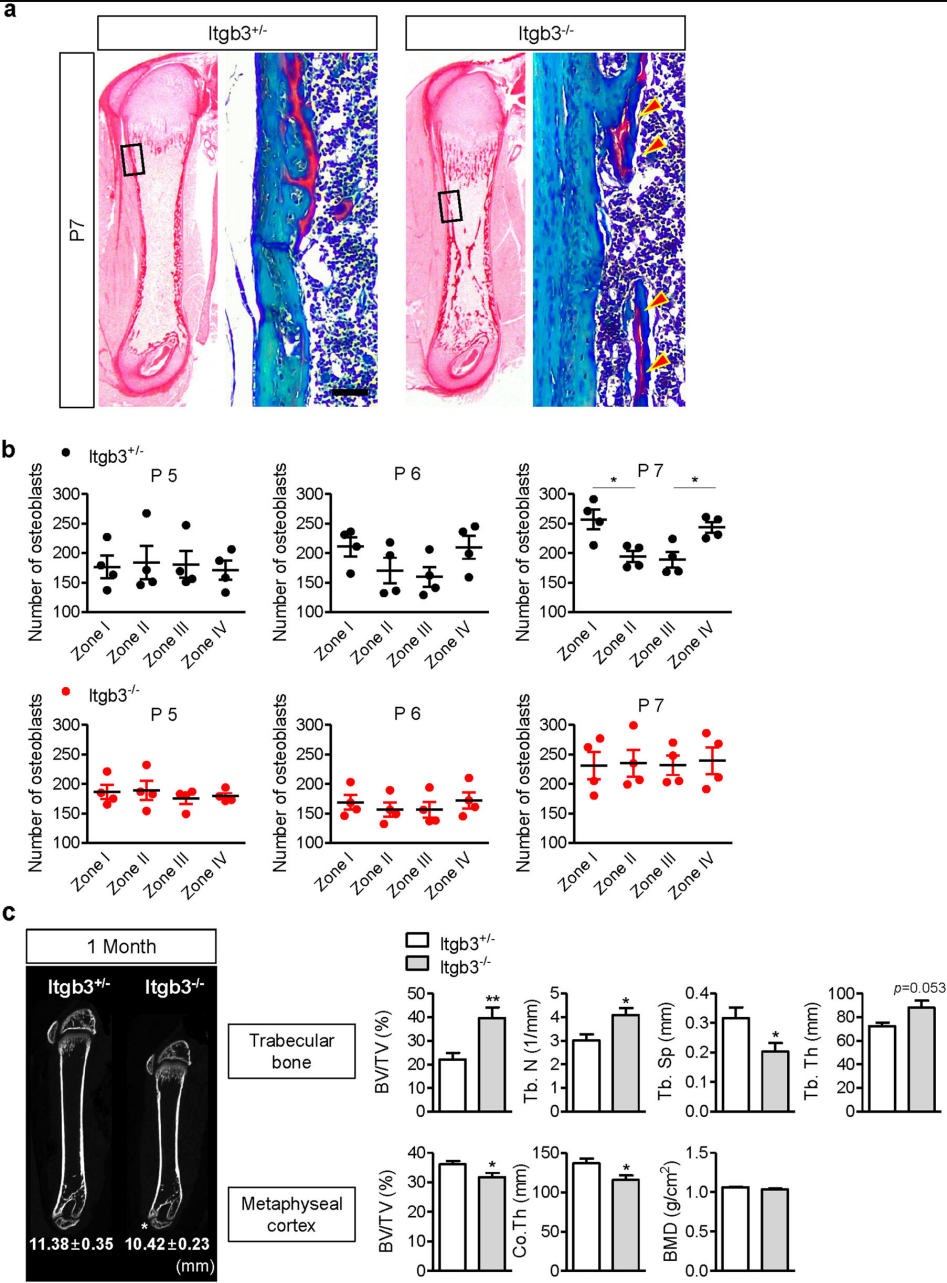
Corticalization is controlled by integrin β3. **a** Picrosirius red staining and Safranin O staining of femora of 7-day-old male littermates. Yellow arrowheads indicate the cartilaginous portion of the trabecular bone. **b** The number of osteoblasts according to zone at P5, P6, and P7. Values are presented as the mean ± SEM (*n* = 5). *p < 0.05, zone I versus zone II or zone III versus zone IV. **c** Morphometric indices in trabecular bone or metaphyseal cortex of the distal femur at 1 month of age. Femur length was measured from the proximal end of the femur to the distal end using micro-CT. Values are presented as the mean ± SEM (*n* = 5). *p < 0.05, **p < 0.01 versus controls. Scale bar, 50 µm

To investigate whether integrin β3, which is modulated by Osx, affects corticalization, we analyzed femora from *Itgb3*-null mice. Interestingly, in the femora of *Itgb3*-null mice, the inner and the outer cortical bone was separated, and the trabecular bone volume of the central spongiosa was increased at P7 with no observable growth plate differences (Fig. 4a, Fig. S4). Furthermore, these bone phenotypes were also observed in tibia. However, the detachment of bone from cortical bone vanished gradually with age (Fig. 4c). To determine whether the bone separation was a result of a reduction in bone resorption in the bone collar (intramembranous ossification)^36^ or detachment of the trabecular bone (endochondral ossification), we performed Safranin O staining. Separated bone was stained red (Fig. 4a). This showed that bone formed by endochondral ossification did not coalesce with the bone collar and was separated in *Itgb3*-null mice.

As osteoblasts did not migrate to the peripheral spongiosa in Col-OMT mice, the number of osteoblasts in peripheral zones was not changed compared to the central zone in *Itgb3*-null mice at P7 (Fig. 4b). At 1 month of age, femoral length decreased significantly in *Itgb3-*null mice compared to *Itgb3*^*+/−*^ mice (Fig. 4c). In the μCT analysis of trabecular bone at 1 month of age, the trabecular number and the bone volume to total tissue volume ratio (BV/TV) were higher in *Itgb3-*null mice than in *Itgb3*^*+/−*^ mice. Trabecular separation (Tb.Sp) was significantly lower in *Itgb3-*null mice. On the other hand, BV/TV and cortical thickness were significantly decreased in the metaphyseal cortical bone of the distal femur in *Itgb3-*null mice (Fig. 4c). As in Col-OMT mice, *Itgb3-*null mice exhibited an abnormal distribution of osteoblasts at the beginning of corticalization and decreased limb length and delayed corticalization at 1 month. However, unlike in Col-OMT mice, cortical BMD was not changed in *Itgb3-*null mice compared to *Itgb3*^*+/−*^ mice. These results suggest that Osx regulates bone mineralization and corticalization, whereas integrin β3 controls corticalization without affecting bone mineralization.

## Discussion

**Fig. 5:**
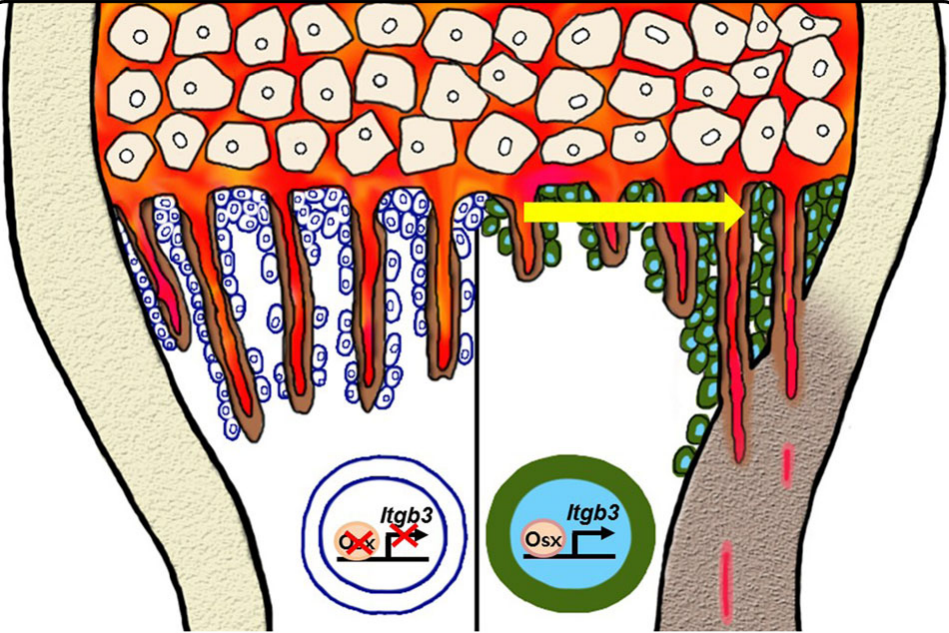
Proposed schematic of the role of osterix in the control of corticalization. At about postnatal 7 days, osterix directly controls the expression of integrin β3, which regulates corticalization for longitudinal bone growth

This study showed that corticalization affecting longitudinal bone growth takes place at postnatal day 7 with the migration of osteoblasts to the peripheral spongiosa. Additionally, the regulation of integrin β3 by osterix in osteoblasts plays an important role in corticalization (Fig. 5).

Longitudinal bone growth is largely determined by the rate of growth plate chondrogenesis^9^. However, taking into consideration that corticalization (the process by which the bone formed in the growth plate is fused to the metaphyseal cortical bone) is the last step in the longitudinal bone growth process^6^ and that corticalization is modulated by Osx in osteoblasts (osteoblast differentiation), osteoblasts play a role in limb growth (Fig. 5). A large meta-analysis genome-wide association study showed that genes in loci associated with the regulation of normal variation in adult human height tend to be highly expressed in osteoblast-related tissues as well as in chondrocytes^37^. Furthermore, many studies in knockout mouse models have shown that osteoblasts affect growth retardation. Fibrillins, the major structural components of extracellular microfibrils, were observed in the endosteal surface of trabecular bone and are secreted from osteoblasts^38^. *Fibrillin-1* and *fibrillin-2* knockout mice exhibited differences in bone length compared to controls without growth plate anomalies^12^. Bone sialoprotein (BSP), a SIBLING protein (small, integrin-binding ligand N-linked glycoprotein), is a component of mineralized tissues such as bone. Femoral length was decreased in BSP knockout mice compared to control mice with no growth plate differences or nutritional defects^10^. Similar to *Itgb3*-null mice, BSP knockout mice displayed a high trabecular bone mass. In particular, osteocyte-specific knock-in mice expressing a dominant-active *β-catenin* in osteocytes failed to show longitudinal limb growth despite a marked increase in trabecular bone^13^. These mice also did not display differences in growth plate thickness or serum phosphate levels. These results support the finding that osteoblasts and osteocytes regulate longitudinal limb growth without affecting the growth plate.

In this study, osteoblasts in the primary spongiosa migrated to the peripheral spongiosa and formed clusters (cell−cell) for coalescence in cortical bone (cell−matrix) by postnatal (mechanical stimulation) day 10 (Figs. 1 and 2). These changes indicate a role for integrin signaling. Therefore, we analyzed integrin signaling in Col-OMT mice, which exhibited delayed corticalization.

We studied integrin signaling in Col-OMT mice for an additional reason. SIBLINGs are a family of five integrin-binding proteins (osteopontin (OPN), bone sialoprotein (BSP), dentin matrix protein 1 (DMP1), dentin sialophosphoprotein (DSPP), and matrix extracellular phosphoglycoprotein (MEPE)) that modulate cell adhesion by interacting with integrins^39^. Notably, DMP1 binds to the integrin αvβ3 and stimulates phosphorylation of FA kinases^40^. Because DMP1 is significantly reduced in *Osx-*knockout mice^21,41^, we investigated whether disruption of *Osx* affected integrin signaling. In control mice, DMP1 expression was increased in trabecular bone at P6 compared to P4. In contrast, DMP1 expression did not change in Col-OMT mice (data not shown). The expression of an Osx-modulated ligand (DMP1) also partially affected the induction of integrin signaling.

In the present study, we showed that integrin β3 signaling is involved in the migration of osteoblasts to the peripheral zone at the beginning of corticalization. Considering the mechanism by which integrin β3 signaling leads to the migration of osteoblasts to the peripheral zone, we speculated that mechanical forces or osteoblast differentiation stimuli would be increased in the peripheral zone compared to the central zone. Furthermore, osteoblasts could be recruited and forced to cluster in the peripheral zone due to mechanical loading-induced secretion of cytokines or growth factors from osteocytes in cortical bone^42,43^. Second, ECMs (SIBLINGs) in the metaphyseal cortex, which is formed through intramembranous ossification in the bone collar, are thought to trigger the migration of osteoblasts to the peripheral zone through integrin β3.

The increase in trabecular bone volume could be affected by osteoclast function in *Itgb3-*null mice. β3^−/−^ osteoclasts cultured from bone marrow macrophages were defective in their resorption capacity and showed abnormal cytoskeletal morphology^36^. However, abnormal distribution of osteoblasts and the detachment of trabecular bone was not modulated by osteoclast function. Furthermore, because osteoblasts in the primary spongiosa expressed integrin β3 signaling at P6, integrin β3 signaling might conceivably play a role in osteoblast functioning (Fig. 3). Early studies indicated that several integrins interact with ECM components, control osteoblast functions and are involved in bone formation^26,44,45^. In addition, some clinical studies have suggested that delayed corticalization can bring about an increase in trabecular bone volume. Cortical thickness and cortical volumetric BMD were significantly lower in the distal radius and distal tibia of males compared to females, despite increased trabecular BV/TV and trabecular thickness during puberty^6^. Reports have suggested that this transitory deficit in cortical bone formation results from a delay in trabeculae corticalization. Based on these reports, it has been suggested that the increase in trabecular bone volume is partially influenced by integrin β3 signaling in osteoblasts due to delayed corticalization.

Glanzmann thrombasthenia (GT), a rare hereditary autosomal-recessive bleeding disorder, is associated with integrin β3^27^. Although GT bone phenotypes are reported less frequently than the blood disorders associated with this disease (mainly focused on platelet function), several reports showed that patients with GT exhibited short stature (below the tenth percentile)^46,47^. In particular, patients with αIIbβ3 integrin activation failure due to a point mutation in *KINDLIN3* displayed heights within only the third percentile. Further, osteopetrosis was observed in bone proximal to the growth plate at 7 months of age^48^. Malinin et al. suggested that integrin activation may be involved in osteogenesis as well as in bone resorption, as subjects’ mesenchymal stem cells produced substantially higher amounts of bone. Furthermore, allogenic bone marrow transplantation (BMT) resolved the macroscopic bone density defects, and bone production by the mesenchymal stem cells also reverted to control levels after BMT. Our results and those of previous reports suggest that integrin β3 plays a role in longitudinal bone growth.

In conclusion, osteoblasts contribute to longitudinal limb growth through corticalization after birth. Corticalization is mediated by integrin β3 signaling in differentiated osteoblasts. Because differences in osteoblast function can affect normal variations in height, the control of osteoblast function during corticalization may help in the treatment of short stature.

## Electronic supplementary material

Supplementary information

Figure S1

Figure S2

Figure S3

Figure S4

## References

[1] van der Eerden BC, Karperien M, Wit JM (2003). Systemic and local regulation of the growth plate. Endocr. Rev..

[2] Shiang R (1994). Mutations in the transmembrane domain of FGFR3 cause the most common genetic form of dwarfism, achondroplasia. Cell.

[3] St-Jacques B, Hammerschmidt M, McMahon AP (1999). Indian hedgehog signaling regulates proliferation and differentiation of chondrocytes and is essential for bone formation. Genes Dev..

[4] Kronenberg HM (2003). Developmental regulation of the growth plate. Nature.

[5] Long F, Ornitz DM (2013). Development of the endochondral skeleton. Cold Spring Harb. Perspect. Biol..

[6] Wang Q (2010). Rapid growth produces transient cortical weakness: a risk factor for metaphyseal fractures during puberty. J. Bone Miner. Res..

[7] Wang Q, Ghasem-Zadeh A, Wang XF, Iuliano-Burns S, Seeman E (2011). Trabecular bone of growth plate origin influences both trabecular and cortical morphology in adulthood. J. Bone Miner. Res..

[8] Bala Y (2015). Trabecular and cortical microstructure and fragility of the distal radius in women. J. Bone Miner. Res..

[9] Baron J (2015). Short and tall stature: a new paradigm emerges. Nat. Rev. Endocrinol..

[10] Malaval L (2008). Bone sialoprotein plays a functional role in bone formation and osteoclastogenesis. J. Exp. Med..

[11] Baek WY (2009). Positive regulation of adult bone formation by osteoblast-specific transcription factor osterix. J. Bone Miner. Res..

[12] Arteaga-Solis E (2011). Material and mechanical properties of bones deficient for fibrillin-1 or fibrillin-2 microfibrils. Matrix Biol..

[13] Tu X (2015). Osteocytes mediate the anabolic actions of canonical Wnt/β-catenin signaling in bone. Proc. Natl. Acad. Sci. USA.

[14] Caplan, A. I. & Pechak, D. G . The cellular and molecular embryology of bone formation. In *Bone and Mineral Research* (ed. Peck, W. A.) 117–183 (Elsevier Science Publishers, New York, NY, 1987).

[15] Karsenty G, Wagner EF (2002). Reaching a genetic and molecular understanding of skeletal development. Dev. Cell.

[16] Maes C (2010). Osteoblast precursors, but not mature osteoblasts, move into developing and fractured bones along with invading blood vessels. Dev. Cell.

[17] Yang L, Tsang KY, Tang HC, Chan D, Cheah KS (2014). Hypertrophic chondrocytes can become osteoblasts and osteocytes in endochondral bone formation. Proc. Natl. Acad. Sci. USA.

[18] Cadet ER (2003). Mechanisms responsible for longitudinal growth of the cortex: coalescence of trabecular bone into cortical bone. J. Bone Jt. Surg. Am..

[19] Tanck E (2006). Cortical bone development under the growth plate is regulated by mechanical load transfer. J. Anat..

[20] Nakashima K (2002). The novel zinc finger-containing transcription factor osterix is required for osteoblast differentiation and bone formation. Cell.

[21] Zhou X (2010). Multiple functions of Osterix are required for bone growth and homeostasis in postnatal mice. Proc. Natl. Acad. Sci. USA.

[22] Lee SJ, Lee EH, Park SY, Kim JE (2017). Induction of fibrillin-2 and periostin expression in Osterix-knockdown MC3T3-E1 cells. Gene.

[23] Marie PJ, Haÿ E, Saidak Z (2014). Integrin and cadherin signaling in bone: role and potential therapeutic targets. Trends Endocrinol. Metab..

[24] Weyts FA, Li YS, van Leeuwen J, Weinans H, Chien S (2002). ERK activation and alpha v beta 3 integrin signaling through Shc recruitment in response to mechanical stimulation in human osteoblasts. J. Cell. Biochem..

[25] Thompson WR, Rubin CT, Rubin J (2012). Mechanical regulation of signaling pathways in bone. Gene.

[26] Marie PJ (2013). Targeting integrins to promote bone formation and repair. Nat. Rev. Endocrinol..

[27] Bouvard D, Pouwels J, De Franceschi N, Ivaska J (2013). Integrin inactivators: balancing cellular functions in vitro and in vivo. Nat. Rev. Mol. Cell Biol..

[28] Hodivala-Dilke KM (1999). Beta3-integrin-deficient mice are a model for Glanzmann thrombasthenia showing placental defects and reduced survival. J. Clin. Invest..

[29] Hynes RO (2002). Integrins: bidirectional, allosteric signaling machines. Cell.

[30] DeMali KA, Sun X, Bui GA (2014). Force transmission at cell−cell and cell−matrix adhesions. Biochemistry.

[31] Case LB (2015). Molecular mechanism of vinculin activation and nanoscale spatial organization in focal adhesions. Nat. Cell Biol..

[32] Humphries JD (2007). Vinculin controls focal adhesion formation by direct interactions with talin and actin. J. Cell Biol..

[33] Atherton P, Stutchbury B, Jethwa D, Ballestrem C (2016). Mechanosensitive components of integrin adhesions: role of vinculin. Exp. Cell Res..

[34] Diez G, Auernheimer V, Fabry B, Goldmann WH (2011). Head/tail interaction of vinculin influences cell mechanical behavior. Biochem. Biophys. Res. Commun..

[35] Auernheimer V (2015). Vinculin phosphorylation at residues Y100 and Y1065 is required for cellular force transmission. J. Cell Sci..

[36] McHugh KP (2000). Mice lacking beta3 integrins are osteosclerotic because of dysfunctional osteoclasts. J. Clin. Invest..

[37] Wood AR (2014). Defining the role of common variation in the genomic and biological architecture of adult human height. Nat. Genet..

[38] Kitahama S (2000). Expression of fibrillins and other microfibril-associated proteins in human bone and osteoblast-like cells. Bone.

[39] Bellahcène A, Castronovo V, Ogbureke KU, Fisher LW, Fedarko NS (2008). Small integrin-binding ligand N-linked glycoproteins (SIBLINGs): multifunctional proteins in cancer. Nat. Rev. Cancer.

[40] Wu H (2011). Dentin matrix protein 1 (DMP1) signals via cell surface integrin. J. Biol. Chem..

[41] Bouvard D (2007). Defective osteoblast function in ICAP-1-deficient mice. Development.

[42] Wu AC, Kidd LJ, Cowling NR, Kelly WL, Forwood MR (2014). Osteocyte expression of caspase-3, COX-2, IL-6 and sclerostin are spatially and temporally associated following stress fracture initiation. Bone Rep..

[43] Pathak JL (2015). Mechanical loading reduces inflammation-induced human osteocyte-to-osteoclast communication. Calcif. Tissue Int..

[44] Moon YJ (2016). Smad4 controls bone homeostasis through regulation of osteoblast/osteocyte viability. Exp. Mol. Med..

[45] Brunner M (2011). Osteoblast mineralization requires beta1 integrin/ICAP-1-dependent fibronectin deposition. J. Cell Biol..

[46] Hathaway WE (1971). Bleeding disorders due to platelet dysfunction. Am. J. Dis. Child..

[47] Toygar HU, Guzeldemir E (2007). Excessive gingival bleeding in two patients with Glanzmann thrombasthenia. J. Periodontol..

[48] Malinin NL (2009). A point mutation in KINDLIN3 ablates activation of three integrin subfamilies in humans. Nat. Med..

