# Supplementary material for: Osterix regulates corticalization for longitudinal bone growth via integrin β3 expression
Source: Exp Mol Med. 2018 Jul 18;50(7):1–11. doi: 10.1038/s12276-018-0119-9 (PMC6052162; doi:10.1038/s12276-018-0119-9)
Supplement: Supplementary file 1 — Supplementary information [file 12276_2018_119_MOESM1_ESM.pdf]

Osterix regulates corticalization for longitudinal bone growth via Integrin β3 expression

Young Jae Moon[1,2], Chi-Young Yun[2], Hwajung Choi[2], Jung Ryul Kim[3,4], Byung-Hyun Park[1], and Eui-Sic Cho[2,*]

[1]Departments of Biochemistry, Chonbuk National University Medical School, Jeonju, Jeonbuk 54896, Republic of Korea

[2]Cluster for Craniofacial Development and Regeneration Research and Institute of Oral Biosciences, Chonbuk National University School of Dentistry, Jeonju, Jeonbuk 54896, Republic of Korea

[3]Departments of Orthopaedic Surgery, Chonbuk National University Medical School, Jeonju, Jeonbuk 54896, Republic of Korea

[4]Research Institute of Clinical Medicine, Chonbuk National University Hospital, Jeonju, Jeonbuk 54907, Republic of Korea

Contents

1. Supplementary Figure Legends
2. Supplementary Table

1. Supplementary Figure Legends

Figure S1 (a) 3D µCT reconstruction of femur to show the section of femur. (b) Hematoxylin and eosin staining of femur at P7.

Figure S2 Distal femur of *Col 1a1-Cre: R26R* mice were stained for β-gal activity. Black bar, 100 µm.

Figure S3 Disruption of Osterix in osteoblasts. Osterix immunostaining of femur tissue for osteoblasts from control and Col-OMT. (a) central trabecular lesion (b) peripheral trabecular lesion. Scale bar, 100 µm**,** GP, growth plate, C, cortex.

Figure S4 The growth plate phenotype in *Itgb3*$^{+/-}$ mice and *Itgb3* null mice. Scale bar, 25 µm.

2. Supplementary Table

Table S1 Sequence and accession numbers for primers (forward, FOR; reverse, REV) used in real-time RT-PCR.

Gene	Sequence for primers
Itgav	FOR: CGGGTCCCGAGGGAAGTTA<br>REV: TGGATGAGCATTCACATTTGAGA
Itga2	FOR: CGGCAGAGATCGATACACATAACC<br>REV: CCTATGATAACCCCTGTCGGTACTT
Itga4	FOR: TGCGGAAGCGAGGTGCAGAC<br>REV: CTTTCTGATCCCGCATCTGT
Itga5	FOR: TGCAGTGGTTCGGAGCAAC<br>REV: TTTTCTGTGCGCCAGCTATAC
Itga8	FOR: CGTCTCTGTTGCGTCTCGG<br>REV: ATCCAAGTTGAACGCCAGACA
Itgb1	FOR: TGGTCAGCAACGCATATCTGG<br>REV: GATCCACAAACCGCAACCT
Itgb3	FOR: GGCGTTGTTGTTGGAGAGTC<br>REV: CTTCAGGTTACATCGGGGTGA
Itgb5	FOR: GAAGTGCCACCTCGTGTGAA<br>REV: GGACCGTGGATTGCCAAAGT
Itgb6	FOR: ACTGTCTTGGTAGGTAACCTTCA<br>REV: TGGCTTCATAGCAGTTGCCAC
Itgb8	FOR: TGCATGTTGTAACGTCAAGTGA<br>REV: GATGCTGACACATCAACCAGATA
