# Supplementary figures and images for: Osterix regulates corticalization for longitudinal bone growth via integrin β3 expression
Source: Exp Mol Med. 2018 Jul 18;50(7):1–11. doi: 10.1038/s12276-018-0119-9 (PMC6052162; doi:10.1038/s12276-018-0119-9)

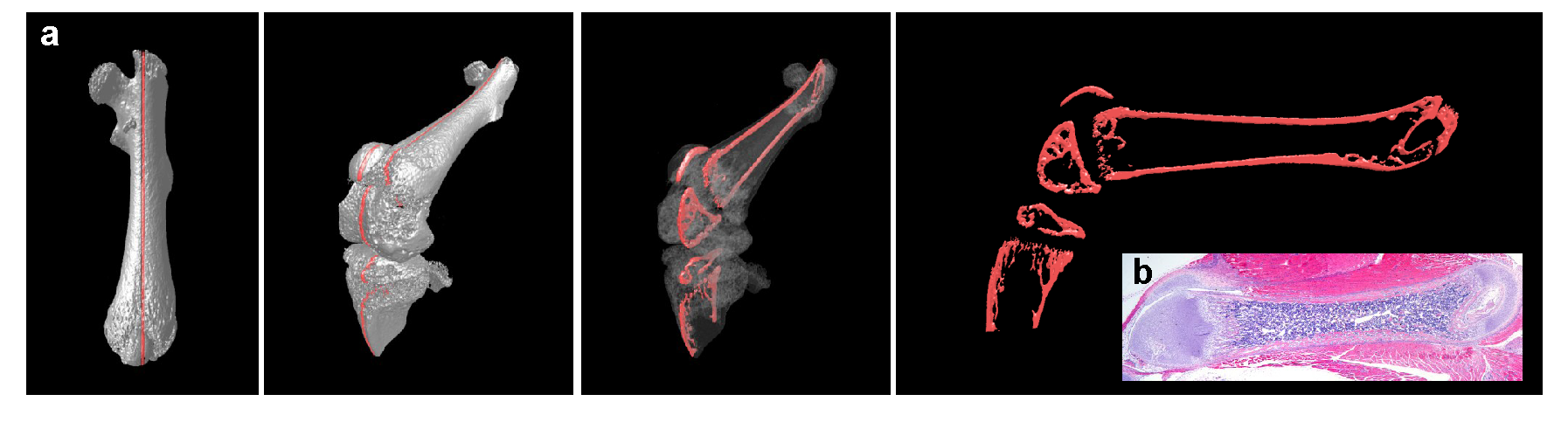

Supplement: Supplementary file 2 — Figure S1 [file 12276_2018_119_MOESM2_ESM.tif]

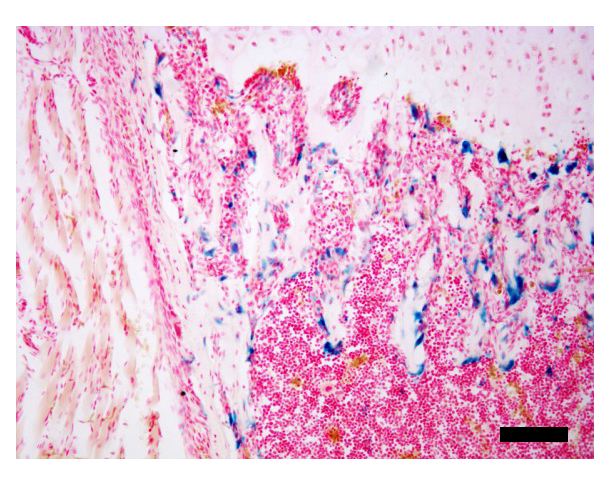

Supplement: Supplementary file 3 — Figure S2 [file 12276_2018_119_MOESM3_ESM.tif]

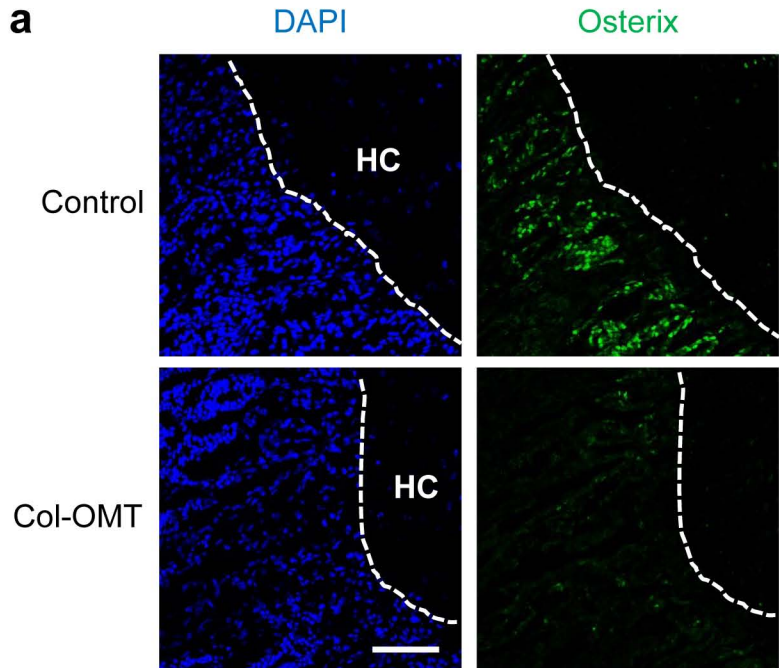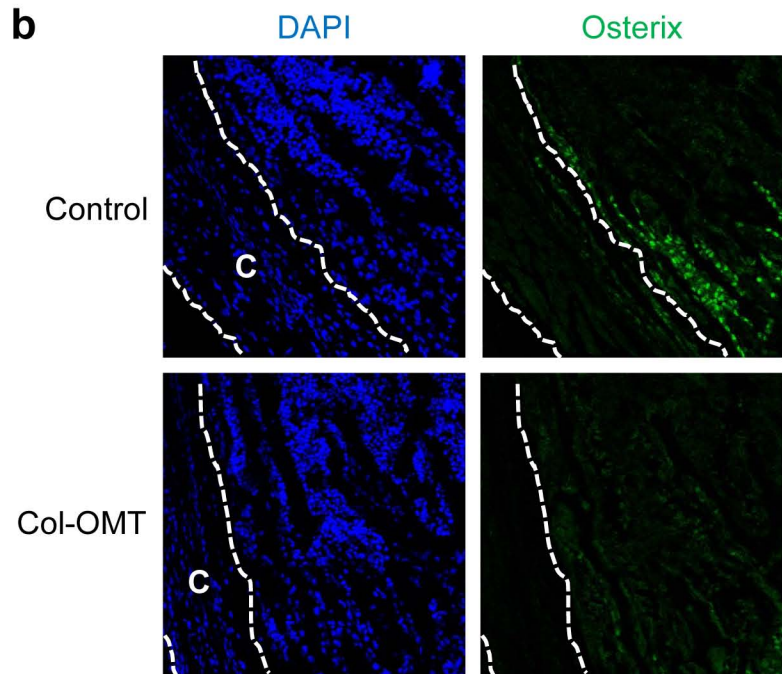

Supplement: Supplementary file 4 — Figure S3 [file 12276_2018_119_MOESM4_ESM.pdf]

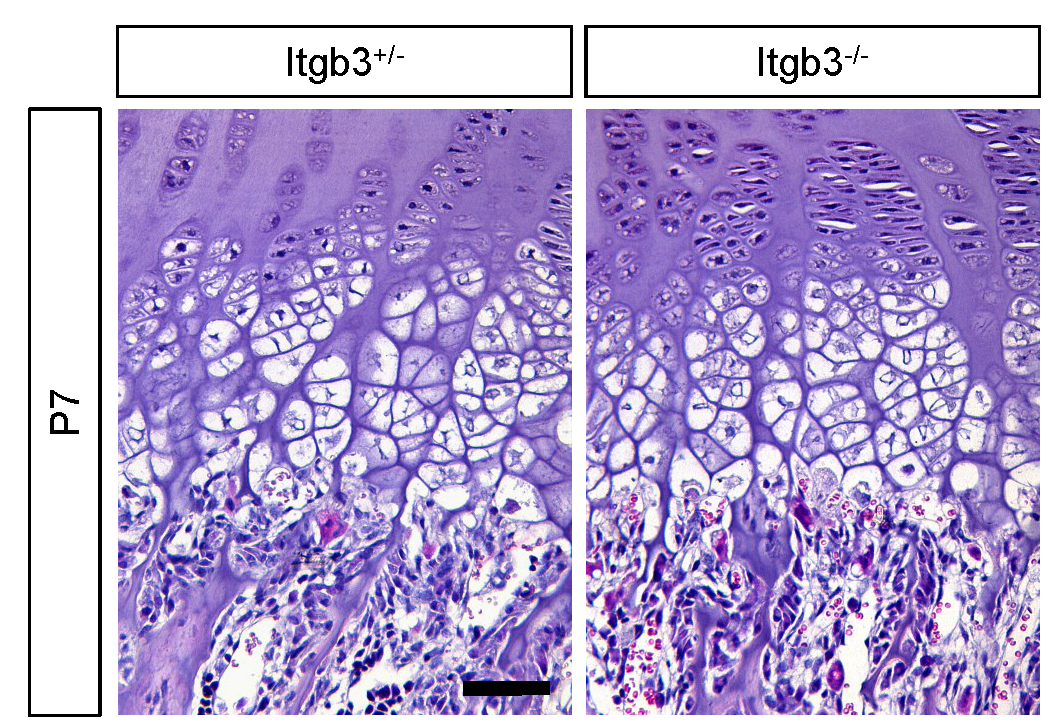

Supplement: Supplementary file 5 — Figure S4 [file 12276_2018_119_MOESM5_ESM.tif]
